# Typification of the Economically Important Species *Thyreophagus entomophagus* (Acari: Astigmata: Acaridae) Used for the Industrial Production of Predatory Mites: The Designation of a Neotype with Detailed Morphological and DNA Sequence Data †

**DOI:** 10.3390/ani15030357

**Published:** 2025-01-26

**Authors:** Pavel B. Klimov, Vasiliy B. Kolesnikov, Alexander A. Khaustov, Vladimir A. Khaustov, Jonas Merckx, Marcus V. A. Duarte, Dominiek Vangansbeke, Ilse Geudens, Almir Pepato

**Affiliations:** 1 Lilly Hall of Life Sciences, Purdue University, G-225, 915 W State St., West Lafayette, IN 47907, USA; 2 X-Bio Institute, Tyumen State University, 10 Semakova Str., 625003 Tyumen, Russia; jukoman@yandex.ru (V.B.K.); alkhaustov@mail.ru (A.A.K.); kh4ustov93@yandex.ru (V.A.K.); 3 Papanin Institute for Biology of Inland Waters, Russian Academy of Sciences, 152742 Borok, Russia; 4 Biobest Sustainable Crop Management, R&D, 2260 Westerlo, Belgium; jonas.merckx@biobestgroup.com (J.M.); marcus.duarte@biobestgroup.com (M.V.A.D.); dominiek.vangansbeke@ugent.be (D.V.); ilse.geudens@biobestgroup.com (I.G.); 5 Biodiversity Inventory for Conservation NPO (BINCO), Walmersumstraat 44, 3380 Glabbeek, Belgium; 6 Laboratório de Sistemática eEvolução de Ácaros Acariformes, Instituto de Ciências Biológicas, Departamento de Zoologia, Universidade Federal de Minas Gerais, Av. Antonio Carlos, 6627, Pampulha, Belo Horizonte 31270-901, Brazil; apepato@gmail.com

**Keywords:** astigmatid mites, prey mites, predatory mites, morphology, redescription, laboratory culture

## Abstract

The mite *Thyreophagus entomophagus* is a cosmopolitan species of significant economic importance in biocontrol applications, serving as a factitious prey for the mass rearing of predatory mites. This species has been reported from a variety of habitats. However, the taxonomic reliability of its name is questionable due to inconsistencies in historical species identifications, the absence of type specimens, and misidentified GenBank sequences. Here, to address these issues and to standardize the nomenclature, we redescribe *Thyreophagus entomophagus* based on a commercial culture with known COX1 barcoding sequence data and designate a neotype from this culture. As part of delimiting the species boundaries of *Th. entomophagus*, the question of whether this species forms heteromorphic deutonymphs is particularly important. While the literature suggests that most populations lack them, at least one population in Germany has been reported to produce heteromorphic deutonymphs. However, after careful examination, we identified this population as a new species, *Thyreophagus holda*, indicating that previous identifications of this population as *Th. entomophagus* were incorrect. The absence of the heteromorphic deutonymphal stage is a beneficial trait for mass production, as it simplifies the life cycle by eliminating the energetically costly heteromorphic deutonymph. Our preliminary molecular phylogenetic analyses of *Th. entomophagus* and other species of *Thyreophagus* indicate that the loss of heteromorphic deutonymphs and the emergence of asexual reproduction (another beneficial trait for mass production) are derived traits that arose after the divergence of the most recent common ancestor of *Thyreophagus*. These insights enhance our understanding of the evolutionary traits that increase the effectiveness of *Th. entomophagus* and related species in biocontrol settings. Our study points to the need for additional bioprospecting efforts to identify new candidate species for biocontrol that possess both asexual reproduction and the absence of heteromorphic deutonymphs.

## Simple Summary

The mite *Thyreophagus* entomophagus is widely used in agriculture as a food source for breeding predatory mites, which are important for biological pest control. However, the identity of this species has been uncertain due to its incorrect identifications involving both morphology and DNA sequence data. To resolve this, we carefully examined a commercial population, selecting a new type specimen from this population to standardize its name. We also discovered that a population previously thought to belong to this species is actually a new species, *Thyreophagus holda*. These findings clarify that *Th. entomophagus* lacks a specialized life stage (deutonymph) in its life cycle, making it easier and more efficient to mass-produce. Our phylogenetic analysis shows that this trait, along with asexual reproduction, evolved after the origin of the genus *Thyreophagus*. We suggest that these traits—being asexual and lacking the deutonymph stage—are ideal for effective mass production in biological pest control. Our study emphasizes the need to explore more mites with these beneficial traits, which could enhance sustainable agricultural practices and reduce the need for chemical pesticides.

## 1. Introduction

Plant pests, particularly insect pests, pose a significant threat to global agricultural production. While chemical pesticides are effective in controlling these pests, they have adverse environmental and health effects, including the development of pesticide-resistant insect populations. Biological control, which utilizes beneficial insects and mites to prey on pest species, offers a more sustainable alternative. Predatory mites, such as those in the family Phytoseiidae, have shown promise in controlling pests like thrips, whiteflies, and red spider mites [1,2,3,4]. The mass rearing of these beneficial insects and mites is important for their widespread application in agricultural settings. While early rearing systems relied on natural food sources, recent technological advances have focused on using factitious prey mites, such as stored product mites, to provide a consistent and controlled food supply for predators [1,2,3,4]. Successful mass rearing of beneficial insects and mites is essential for the continued development and implementation of biological control strategies, reducing reliance on chemical pesticides, and promoting more sustainable agricultural practices.

*Thyreophagus entomophagus* (Laboulbène and Robin, 1862) (Acaridae) has gained popularity as a factitious prey mite due to its favorable characteristics, such as ease of consumption by predators and being less allergenic and less harmful as a stored food pest compared to other species like *Tyrophagus putrescentiae* (Schrank, 1781) [1,2,3,4]. Currently, *Th. entomophagus* is widely used in the mass production of predatory mites globally [5,6,7].

Historically, the specific name *Thyreophagus entomophagus* was proposed as *Acarus entomophagus* by Laboulbène in 1852 [8]; however, this name was a nomen nudum and not compliant with ICZN rules. An ICZN-compliant description was made in 1862 under the name *Tyroglyphus entomophagus* by Laboulbène and Robin for mites damaging entomological collections in Southern France [9]. In 1874, Rondani established a new genus, *Thyreophagus*, to include this species, leading to the currently used binomen *Thyreophagus entomophagus*. Since the type material of *Th. entomophagus* has been lost [10], it was redescribed from adults collected in the Birmingham region of the United Kingdom, without proposing a neotype by A. Fain in 1982 [10]. This species concept continues to be widely used today, however, deutonymphs remained unknown. Phoretic deutonymphs of *Th. entomophagus* were later described from a culture started from specimens collected in a sparrow nest in Dahlem, Berlin, Germany [11], although adults from this German population have never been described. Remarkably, industrially reared populations of *Th. entomophagus* never produce heteromorphic deutonymphs. This suggests that either cryptic species are involved (one can produce deutonymphs while the other cannot), or some populations of *Th. entomophagus* may have permanently lost the ability to produce heteromorphic deutonymphs. Furthermore (personal observations), GenBank has two mitochondrial COX1 sequences identified as *Th. entomophagus* from China (NC_066986) and Russia (OR640974) that differ by a 21.16% COX1 K2P distance [7], indicating that these samples represent two different species. These findings suggest that the taxonomic status of the economically important species *Th. entomophagus* is uncertain in terms of both morphology and genetics. There is an urgent need to standardize the usage of the name to ensure taxonomic stability and scientific repeatability of research that uses the name *Thyreophagus entomophagus*.

In this study, we carefully examine the German deutonymph-producing population of *Thyreophagus entomophagus* to determine if there are morphological differences compared to a commercial population that never forms heteromorphic deutonymphs and compare it with specimens from Birmingham region (United Kingdom) described in Fain [10]. Additionally, we provide a detailed morphological description of the commercial population, supported by COX1 sequences and notes on the phylogeny of *Thyreophagus*. Since the German populations were morphologically distinct from specimens in the commercial culture and the UK population, we propose standardizing the use of the name *Thyreophagus entomophagus* by designating a neotype based on the commercial population, where both the COX1 sequence and morphology are known.

## 2. Materials and Methods

**Figure 1:**
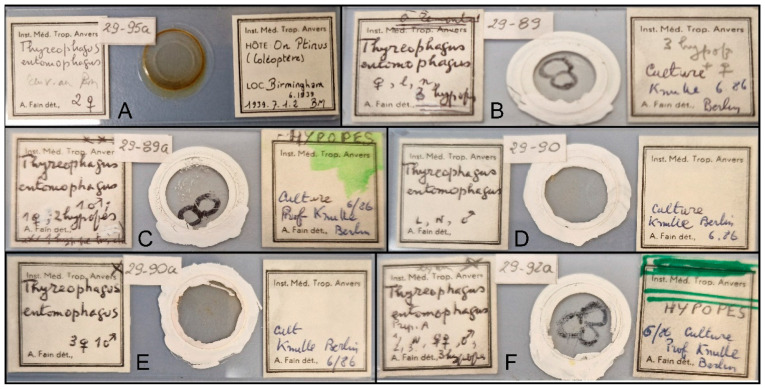
Slides with Fane’s museum specimens examined in this work: (**A**)—specimens *Thyreophagus entomophagus* (Laboulbène and Robin, 1862) from the Birmingham region (United Kingdom); (**B**–**F**)—specimens *Thyreophagus holda* sp. n., collected from a sparrow nest in Dahlem; (**F**)—slide with holotype *Thyreophagus holda* sp. n.

Commercial cultures were obtained from a European biocontrol company on 20 November 2021 and maintained in the lab. The cultures were reared on a mixture of yeast and bran in specialized rearing units. The purity of the cultures was verified through morphological identification of a large number of mites (n = 50). Mites were collected using a camel brush, preserved in 96% ethanol, cleared in 80% lactic acid for 1–2 days, and mounted in Hoyer’s medium, followed by a 7-day drying period at 60 °C [7]. Voucher specimens were deposited at the Tyumen State University Acarological Collection and the Zoological Institute, Russian Academy of Sciences, Saint Petersburg, Russia; the neotype was deposited the Royal Belgian Institute of Natural Sciences, Brussels, Belgium. We also examined specimens collected from a sparrow nest in Dahlem, a suburban district of Berlin, by W. Knülle [11], as well as specimens from the Birmingham region (United Kingdom) described in [10] (Figure 1).

DNA extraction, sequencing, and culture collection information was described previously [7]. For each cultured species, genomic DNA was extracted from 150 females obtained from a pure culture (see above), using a QIAamp DNA Micro kit (Qiagen, Venlo, The Netherlands) with modifications as described here [12]. Illumina sequencing libraries were generated and sequenced commercially on an Illumina NovaSeq 6000 sequencing system. Short Illumina reads were assembled in SPAdes v.3.15.5 [13] as follows: metaspades.py -t 24 -m 240 -1 ${name}_R1_001.fastq -2 ${name}_R2_001.fastq -o ${name}. Full-length cytochrome c oxidase subunit 1 (COX1) sequences were found using a local NCBI BLAST search [14]. Th sequences previously generated by us were used (GenBank accession IDs: OR640973-OR640976). We also used other GenBank species as detailed in the text and figures. We used Mesquite version 3.81 [15] to create sequence alignments, translate nucleotide matrices into protein sequences, and store Nexus matrices for both protein and nucleotide data. Genetic distances were calculated in PAUP v.4a168 [16] as follows: begin paup; dset distance = p; savedist format = tabtext undefined = asterisk file = 1_p_distances.tab; dset distance = k2p; savedist format = tabtext undefined = asterisk file = 2_k2p_distances.tab; end. Phylogenetic trees were inferred in IQ-TREE v. 2.3.4 [17] using amino acid data (command: iqtree2 -s $ipf --seqtype AA -T AUTO -m MFP -alrt 1000 -bb 1000 -safe --prefix $ipf_bn), and nucleotide data (command: iqtree2 -s $ipf -spp partitions.txt -nt 8 -m MFP+MERGE -rcluster 10 -alrt 1000 -bb 1000 -safe --prefix $ipf_bn). Where $ipf is the input file name (a nexus matrix) and $ipf_bn is the basename of $ipf.

Images were captured from multiple focal planes and assembled using Helicon Focus Pro 7.6.4 (algorithm B, occasionally A), with subsequent manual editing (retouching) of any misaligned regions. Partially overlapping images were merged into a full panorama in Adobe Photoshop 22.2.0. Line drawings were created in Photoshop 22.2.0 using microphotographs as the background. The background images were taken using a Euromex Color HD-Ultra camera attached to a Bioptic C-400 microscope (Bioptic, Moscow, Russian Federation) equipped with bright field and differential interference contrast (DIC) optics. Publication-quality microphotographs were captured using an Axio Imager A2 compound microscope (Carl Zeiss, Oberkochen, Germany) equipped with DIC and phase contrast optics, along with an Axiocam 506 color digital camera (Carl Zeiss, Oberkochen, Germany). For scanning electron microscopy (SEM) imaging, alcohol-preserved mites were dried in a JFD 320 freeze dryer (JEOL, Tokyo, Japan), coated with gold, and scanned using a TESCAN Mira3 LMU SEM microscope. Specimens used for SEM were not preserved.

In the descriptions, idiosomal chaetotaxy follows the system outlined in reference [18]; the terminology of coxal setae follows reference [19]. For appendages, chaetotaxy and solenidiotaxy follow Grandjean’s system for palps [20] and legs [21]. Designations of tarsal dorsoapical setae on legs III–IV follow reference [22]. All measurements are given in micrometers (µm).

## 3. Results

### 3.1. GenBank Data Analysis

**Figure 2:**
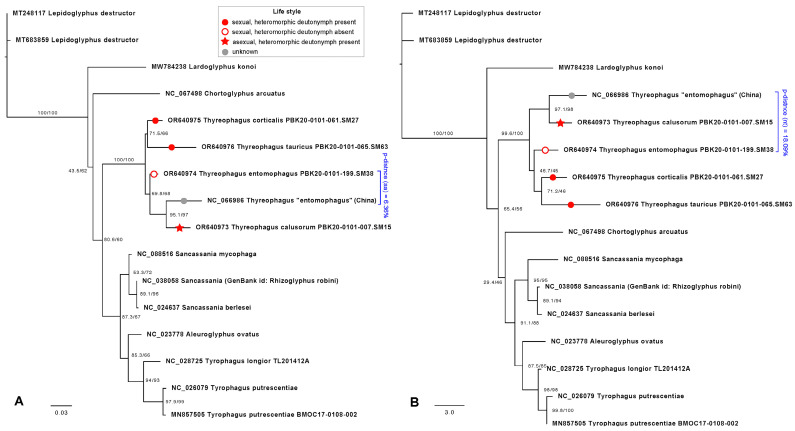
Phylogenetic relationships of species within the genus *Thyreophagus* and outgroups based on cytochrome c oxidase subunit 1 (COX1) sequences, inferred using maximum likelihood analysis in IQ-TREE. SH-aLTR/UFBootstrap values are shown for all nodes. Uncorrected genetic distances (p-distances) are provided for the neotype population (PBK20-0101-199.SM38) vs. Chinese population: (**A**) phylogeny based on protein data using the mtART+I+G4 model; (**B**) phylogeny based on nucleotide data with a codon model.

Maximum likelihood phylogenetic trees were inferred using protein sequences with the mtART+I+G4 model (Figure 2A) and nucleotide sequences with a codon model (Figure 2B). The genus *Thyreophagus* was recovered as monophyletic. The internal relationships within *Thyreophagus* were resolved as two sister groups: (*Th. tauricus*, *Th. corticalis*) and (*Th.* “*entomophagus*” from China [NC 066986], *Th. calusorum*) (Figure 2). However, the placement of *Thyreophagus entomophagus* remained unresolved. In the amino acid phylogeny, *T. entomophagus* was recovered as sister to the *Th*. “*entomophagus*” (China) + *Th. calusorum* clade, whereas in the nucleotide phylogeny, it was sister to the *Th. tauricus* + *Th. corticalis* clade. In both phylogenetic trees, the remaining taxa morphologically assigned to the family Acaridae (*Tyrophagus*, *Sancassania*, and *Aleuroglyphus*) formed a monophyletic group, corresponding to the subfamilies Tyrophaginae and Acotyledonini (Figure 2). However, traditional morphological classifications place *Sancassania* (Acotyledonini) within the subfamily Rhizoglyphinae, which is typically defined by the presence of smooth dorsal setae. Interestingly, *Fagacarus* (Rhizoglyphinae) possesses strongly pectinate setae [23,24], while *Sancassania* exhibits sparse pectinations on some of its setae. These observations suggest that pectinated dorsal setae, a character subject to evolutionary lability, is unreliable for delimiting major groups within Astigmata. In contrast, the presence of the tarsal seta *aa*—although considered plesiomorphic—appears to be a more robust character for defining lineages within Astigmata. In addition, our trees indicate that the GenBank sequence originally identified as *Rhizoglyphus robini* (NC 038058) actually belongs to the genus *Sancassania* (=*Caloglyphus*) (Figure 2). 

### 3.2. Morphological Description

#### 3.2.1. Genus Thyreophagus Rondani, 1874

*Thyreophagus* Rondani, 1874: 67 (=*Moneziella* Berlese, 1897; *Monetiella* Berlese, 1897;

*Monieziella* Berlese, 1897; *Fumouzea* Zachvatkin, 1953; *Michaelopus* Fain and Johnston, 1974).

Type species: *Thyreophagus entomophagus* (Laboulbène and Robin, 1862), by monotypy.

#### 3.2.2. *Thyreophagus entomophagus* (Laboulbène and Robin, 1862)

*Acarus entomophagus* Laboulbène, 1852: LIV (nomen nudum).

*Tyroglyphus entomophagus* Laboulbène and Robin, 1862: 321, Pl. 10 (female, homeomorphic male; types lost [10]).

Material. Cultures were obtained from a European biocontrol company and maintained on a mixture of yeast and bran in specialized rearing units by VAK. Cultures were started on 20 November 2021; specimens were harvested on # COX1 barcoding sequence GenBank Id: OR640974 (culture PBK 20-0101-199.SM38, from which the neotype was designated).

Type material. Neotype (female) same data, deposited at the Royal Belgian Institute of Natural Sciences, Brussels, Belgium.

**Figure 3:**
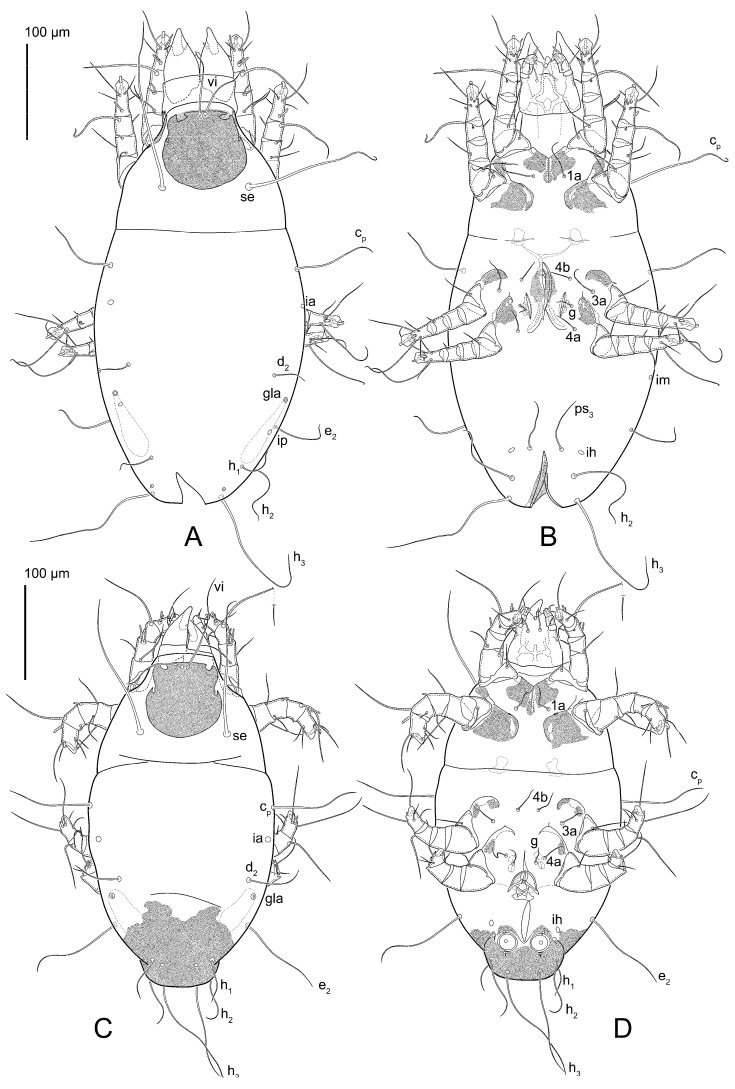
*Thyreophagus entomophagus* (Laboulbène and Robin, 1862), PBK20-0101-199.SM38, neotype (**A**,**B**), not neotype (**C**,**D**): (**A**) female, dorsal view; (**B**) female, ventral view; (**C**) male, dorsal view; (**D**) male, ventral view.

**Figure 4:**
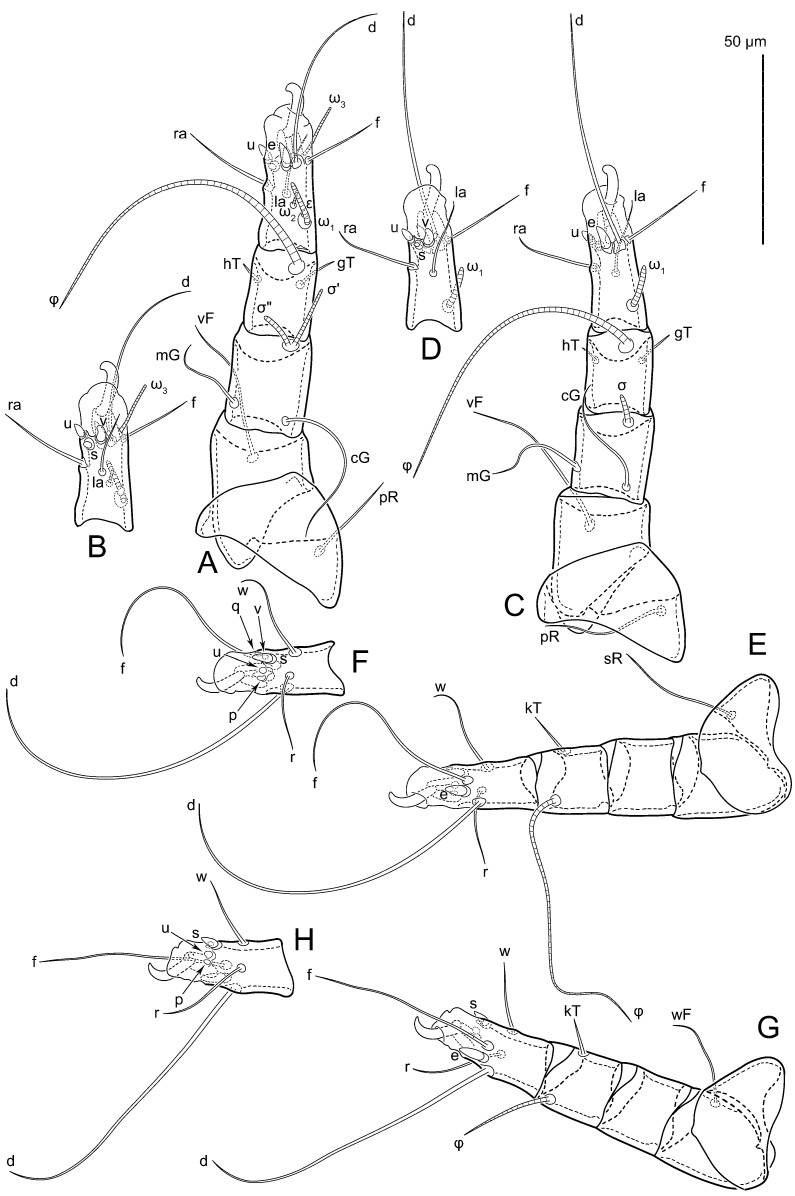
*Thyreophagus entomophagus* (Laboulbène and Robin, 1862), PBK20-0101-199.SM38, female, neotype: (**A**) leg I, dorsal view; (**B**) tarsus I, ventral view; (**C**) leg II, dorsal view; (**D**) tarsus II, ventral view; (**E**) leg III, dorsal view; (**F**) tarsus III, ventral view; (**G**) leg IV, dorsal view; (**H**) tarsus IV, ventral view.

**Figure 5:**
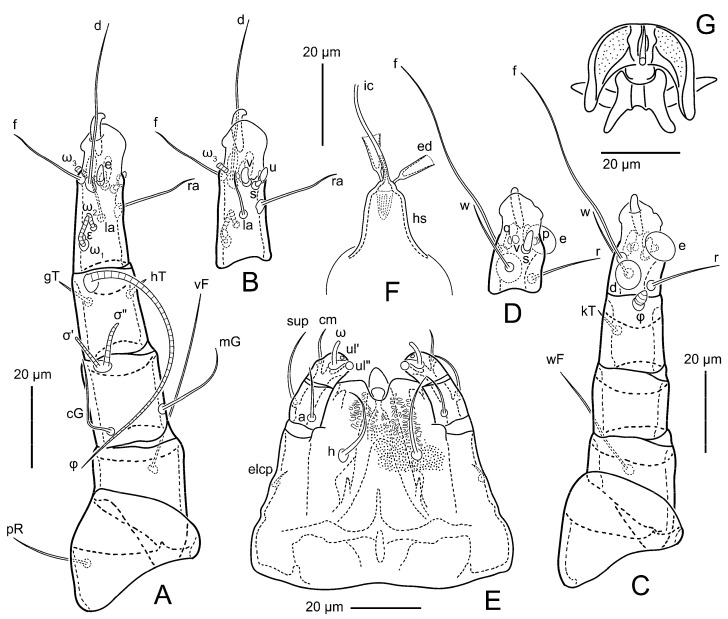
*Thyreophagus entomophagus* (Laboulbène and Robin, 1862), PBK20-0101-199.SM38, neotype (**E**,**F**), non-neotype, but from the same culture (**A**–**D**,**G**): (**A**) male leg I, dorsal view; (**B**) male tarsus I, ventral view; (**C**) male leg IV, dorsal view; (**D**) male tarsus IV, ventral view; (**E**) female gnathosoma, ventral view; (**F**) spermatheca; (**G**) male genitalia.

**Figure 6:**
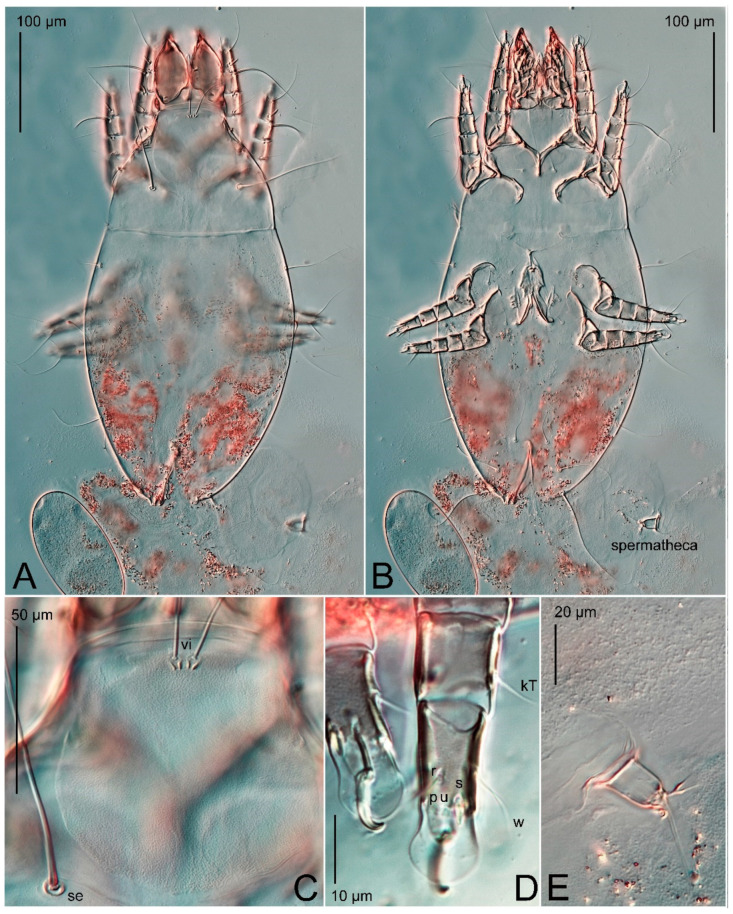
*Thyreophagus entomophagus* (Laboulbène and Robin, 1862), PBK20-0101-199.SM38, female, neotype, DIC images: (**A**) dorsal view; (**B**) ventral view; (**C**) prodorsal shield; (**D**) tarsus III, ventral view; (**E**) spermatheca.

**Figure 7:**
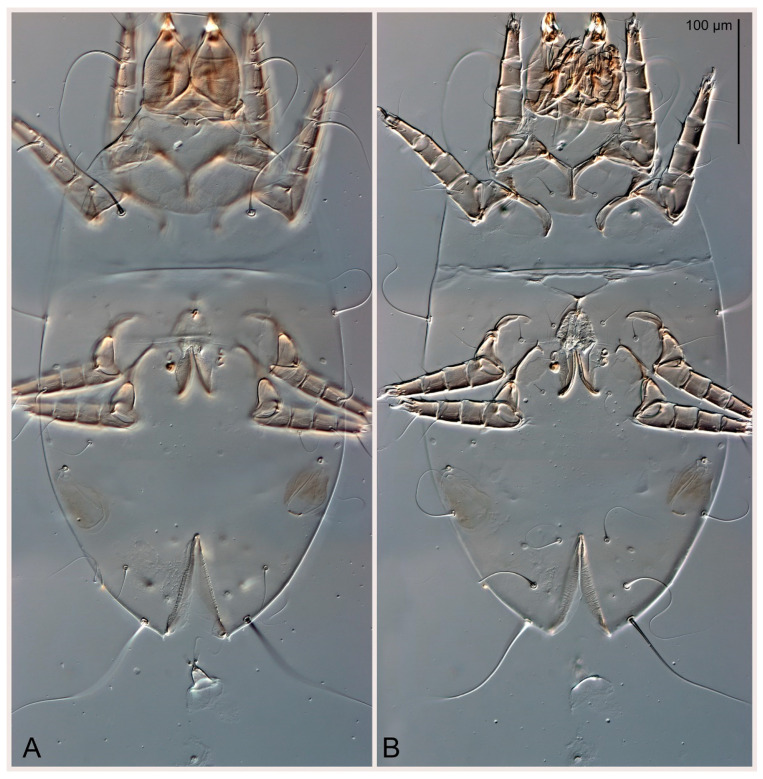
*Thyreophagus entomophagus* (Laboulbène and Robin, 1862), PBK20-0101-199.SM38, female, not neotype, DIC images: (**A**) dorsal view; (**B**) ventral view.

Female (Figure 3A,B, Figure 4, Figure 5E,F, Figure 6, Figure 7, Figure 9D–I, Figure 10 and Figure 18A,D,E,H). Idiosoma slightly elongate, 400–430 × 210–225 (n = 20), 1.9 times longer than wide. Idiosomal cuticle smooth. Subcapitular setae (*h*) long, widened basally; palp tibial setae (*a*), lateral dorsal palp tibial setae (*sup*), dorsal palp tarsal seta (*cm*) filiform; supracoxal seta *elcp* present; terminal palp tarsal solenidion ω short; external part of terminal eupathidium *ul*” dome-shaped; terminal eupathidium *ul*’ small, rounded. Prodorsal shield 73–80 long, 75–84 wide, nearly as long as wide, with setae *vi* (situated at anterior part of shield, alveoli of *vi* noticeably separated—the distance between them almost equal to their width), rounded anterolateral incisions, and elongate midlateral incisions (insertion points of setae *ve*). Prodorsal shield smoothly punctate (with small cells), without distinct lineate pattern (cells may be slightly elongated or rounded in the posterior part) and with several (3–5) curved longitudinal lines in posterior part. Grandjean’s organ (GO) with seven membranous finger-shaped processes. Supracoxal setae (*scx*) smooth, sword-shaped, widened and flattened, tapering at tip. Idiosomal setae (*vi*, *c_p_*, *d*_2_, *e*_2_, *h*_1_, *h*_3_, *ps*_3_) smooth, filiform and short, setae *se* and *h*_2_ twice as long as other idiosomal setae, smooth and filiform; opisthosomal gland openings between setal bases *e*_2_ and *d*_2_. Four pairs of fundamental cupules (*ia*, *im*, *ip* and *ih*) present. With a pair of additional cuticular pores between setae *h*_1_. Ventral idiosoma with four pairs of coxal setae (*1a*, *3a*, *4a* and *4b*) and one pair of genital setae (*g*). Shape of coxal sclerites as in Figure 3B, Figure 6B and Figure 7B. Genital region situated between coxal fields III and IV; genital valves form an inverted Y; epigynal and medial apodemes well developed. Diameter of genital papillae approximately 0.4–0.5 the length of coxal and genital setae. Anal opening terminal. Copulatory tube present, situated anterodorsally to anus, with distinct opening. Inseminatory canal of spermatheca long, slender tube-like, leading from copulatory opening to spermatheca, uniformly wide. Base of spermatheca wide, bell-shaped, with a distinct vestibule. Paired sclerites of efferent ducts elongate, their length approximately 1/2 the length of spermatheca base, with short stem.

Legs short, all segments free. Trochanters I–III each with long, filiform seta, *pR* I–II, *sR* III; trochanter IV without setae. Femoral setation 1-1-0-1; setae *vF* I–II and *wF* IV long, filiform. Genual setation 2-2-0-0; setae *mG* and *cG* I–II long, filiform; seta *nG* III absent. Tibial setation 2-2-1-1; setae *hT* I-II spiniform, shorter than *gT*; setae *gT* I–II and *kT* III–IV elongate, somewhat spiniform. Tarsal setation 8-8-8-8; pretarsi consist of hooked empodial claws attached to short paired condylophores. Tarsi I and II with setae *ra*, *la*, *f* and *d* filiform, *e*, *u*, *v* spiniform, *p* and *q* represented by very small remnants, *s* flattened, button-shaped or minute spiniform (button-shaped in neotype); setae *wa* absent. Tarsi III and IV with setae *f*, *d*, *r*, *w* filiform, *e* and *s* spiniform, *u* and/or *v* flattened, button-shaped, *p* and *q* represented by very small remnants. Solenidion ω_1_ on tarsus I cylindrical, slightly arched, with a slight narrowing before apical widening. Solenidion ω_1_ on tarsus II simple, cylindrical, with clavate apex, nearly straight. Solenidion ω_2_ on tarsus I shorter than ω_1_, cylindrical, with rounded apex, slightly widened distally, situated slightly anteriad ω_1_. Solenidion ω_3_ on tarsus I cylindrical, with rounded tip, subequal to ω_1_, longer than ω_2_. Famulus (ε) of tarsus I wide, spiniform, with broadly rounded apex, widest at middle. Solenidia ϕ of tibiae I–III elongate, tapering, well extending beyond apices of respective tarsi with ambulacra; solenidion ϕ IV shorter than tarsus IV (with ambulacra). Solenidia σ’ and σ” on genu I elongate, tapering, σ” longer than σ’; σ” slightly not reaching bases of ϕ I. Solenidion σ on genu II more than 6–7 times longer than its width, with rounded tip. Solenidion σ of genu III absent.

**Figure 8:**
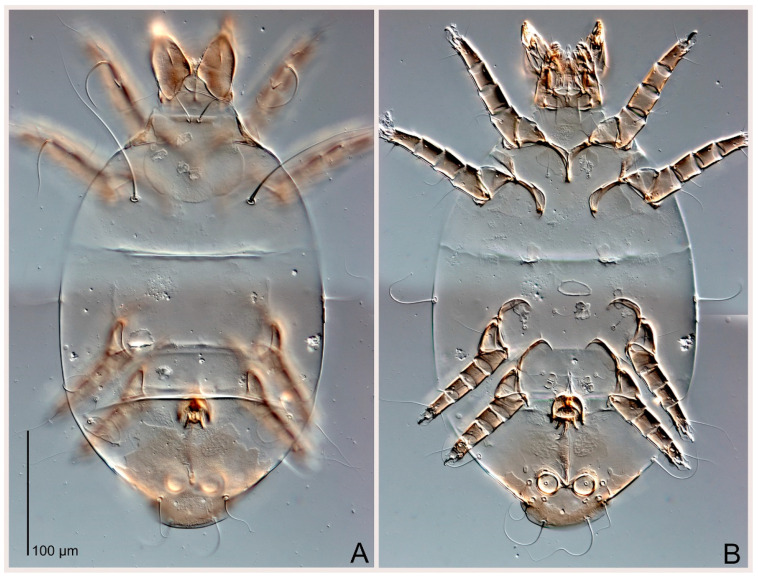
*Thyreophagus entomophagus* (Laboulbène and Robin, 1862), PBK20-0101-199.SM38, male, not neotype, DIC images: (**A**)—dorsal view; (**B**)—ventral view.

**Figure 9:**
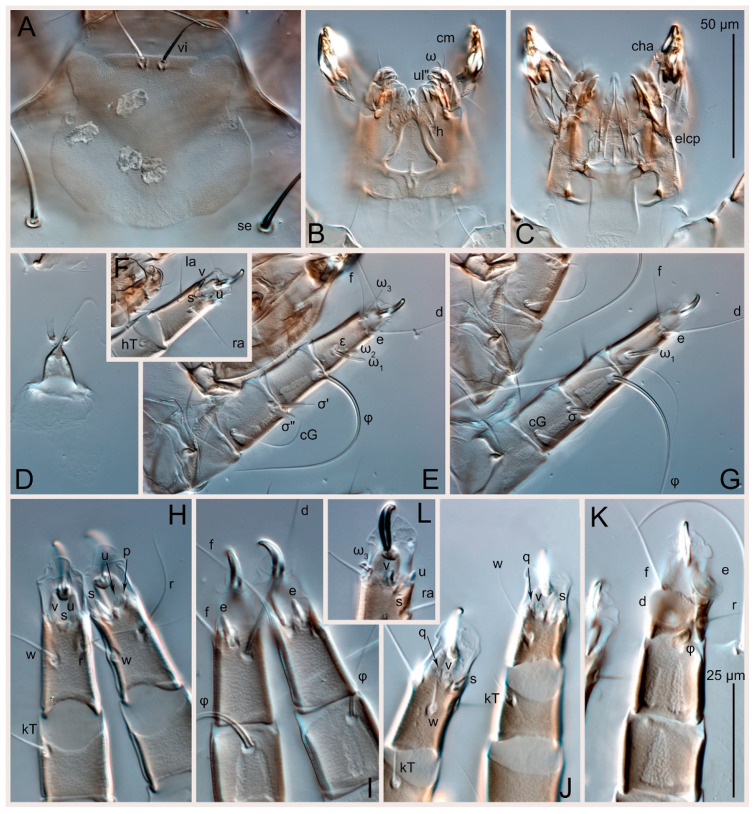
*Thyreophagus entomophagus* (Laboulbène and Robin, 1862), PBK20-0101-199.SM38, not neotype, DIC images: (**A**) male prodorsal shield; (**B**,**C**) male gnathosoma; (**D**) spermatheca; (**E**) female leg I, dorsal view; (**F**) female tarsus I, ventral view; (**G**) female leg I, dorsal view; (**H**) female tarsi III–IV, ventral view; (**I**) female tarsi III–IV, dorsal view; (**J**) male tarsi III–IV, ventral view; (**K**) male tarsus IV, dorsal view; (**L**) male tarsus I, ventral view.

Male (n = 5) (Figure 3C,D, Figure 5A–D,G, Figure 8, Figure 9A–C,L–J and Figure 11). Idiosoma slightly elongate, 300–360 × 180–200, 1.7–1.8 times longer than wide. Idiosomal cuticle smooth. Gnathosoma as in female. Prodorsal shield 73–82 long, 73–80 wide, nearly as long as wide, with setae *vi*, incisions and ornamentation as in female. Grandjean’s organ (GO) and supracoxal seta (*scx*) as in female. All idiosomal setae smooth and filiform, setae *se* longer and wider that other setae; setae *c*_p_, *e*_2_ and *h*_3_ longer than *vi*, *d*_2_, *h*_1_, *h*_2_. Two pairs of fundamental cupules (*ia* and *ih*) present, *im* and *ip* not observed. With a pair of of additional cuticular pores between setae *h*_1_. Opisthonotal shield smoothly punctate; ventral part extends to anal suckers. Ventral idiosoma with four pairs of coxal setae (*1a*, *3a*, *4a* and *4b*) and one pair of genital setae (*g*). Posterior region of idiosoma with a large rounded lobe extending posteriorly (45–48 × 80–86, 1.7–1.8 times longer than wide). Shape of coxal sclerites as in Figure 3D and Figure 8B. Genital region between coxisternal fields IV; genital capsule rounded; aedeagus short, not protruding beyond anterior edge of genital capsule. Diameter of genital papillae approximately 0.4–0.5 the length of coxal and genital setae. Anal suckers rounded in outline. Setae *ps*_1–3_ very short.

**Figure 10:**
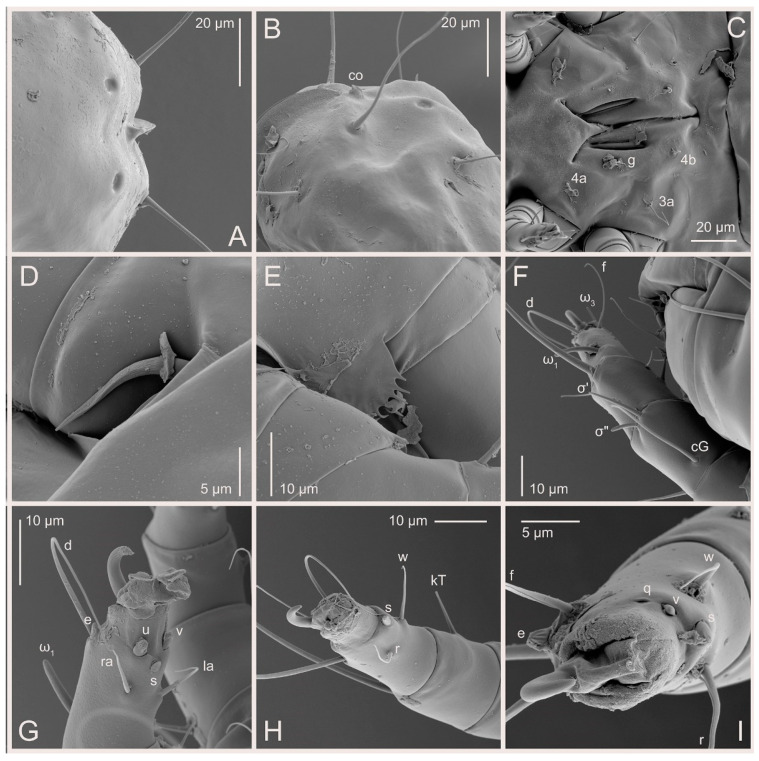
*Thyreophagus entomophagus* (Laboulbène and Robin, 1862), PBK20-0101-199.SM38, female, not neotype, SEM images: (**A**) posterior part of idiosoma, dorsal view; (**B**) posterior part of idiosoma, lateral view; (**C**) genital area, ventral view; (**D**) supracoxal seta; (**E**) Grandjean’s organ; (**F**) leg I, dorsal view; (**G**) tarsus II, ventral view; (**H**,**I**) tarsi III, lateral and ventral views.

**Figure 11:**
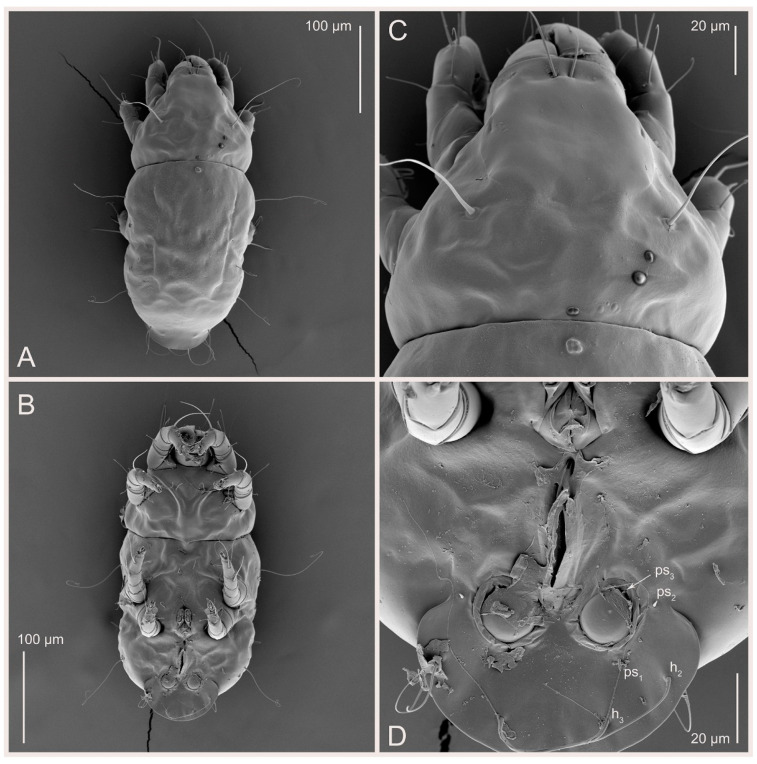
*Thyreophagus entomophagus* (Laboulbène and Robin, 1862), PBK20-0101-199.SM38, male, not neotype, SEM images: (**A**) dorsal view; (**B**) ventral view; (**C**) propodosoma, dorsal view; (**D**) ano-genital region, ventral view.

Legs I–III as in female, except solenidion ω_3_ on tarsus I very short, truncated. Trochanter and genu IV without setae, femur IV with *wF* IV long, filiform, tibia IV with *kT* IV elongate, somewhat spiniform. Tarsus IV with 7 or 8 setae (one *v* or *u* may be absent), of them, *f*, *r*, *w* filiform, *d* and *e* represented by suckers, *s* spiniform, *u* and *v* flattened, button-shaped, *p* and *q* represented by very small remnants. Solenidion ϕ on tibia IV short and wide.

Heteromorphic deutonymph. Absent.

The Birmingham population. The currently used species concept of *Th. entomophagus* was established by A. Fain in 1982 [10], who described specimens from the United Kingdom (Birmingham) without designating a neotype. We re-examined two females from the Birmingham sample and compared them with females from our culture of *Thyreophagus entomophagus* (PBK20-0101-199.SM38). These two were conspecific. Particularly, we note the identical shapes of *u* and *v* III, which are flattened and button-shaped (Figure 18F) and slightly elongated or rounded cells on the posterior part of the prodorsal shield (Figure 18B), without a lineate pattern; A. Fain [10] probably mistook the slightly elongated punctations for a lineate pattern, but they are markedly different from the lineate pattern seen in *Th. holda* sp. n. (Figure 16C). We also note that ventroterminal setae on tarsi III–IV, *u* and *v* III–IV were originally figured by Fain [10] as spiniform, but actually they are flattened and button-shaped structures. The shape of the spermatheca base in the specimens described by A. Fain [10] agrees well with that of all the specimens we studied. In some cases, its width may change, but this is due to deformation during specimen preparation. We were unable to find any noticeable widening of the inseminatory canal, as depicted in A. Fain [10]. Heteromorphic deutonymphs are unknown for the Birmingham sample. 

Diagnosis. Adults of *Th. entomophagus* differ from all other species (except *Th. hobe* Klimov et al., 2023) by the presence of flattened, button-shaped setae *u* and *v* on tarsus III (vs. spiniform in other species). *Thyreophagus entomophagus* differs from *Th. hobe* by the presence of filiform *w* III (minute spiniform in *Th. hobe*) and the presence of *wF* IV (absent in *Th. hobe*). Also, *Th. entomophagus* differs from all other species by the bell-shaped base of spermatheca (vase-shaped, narrow, parallel-sided, broad-arc-shaped, or reduced in other species).

For *Th. entomophagus*, three subspecies have been described: *Th. entomophagus nominalis* Kadzhaya, 1973, *Th. entomophagus ponticus* Kadzhaya, 1973 [25] and *Th. entomophagus italicus* Vacante, 1989 [26]. Subsequently, *Th. entomophagus ponticus* and *Th. entomophagus italicus* were elevated to the rank of species [6]. 

*Thyreophagus ponticus* Kadzhaya, 1973 has a well-developed opisthosomal projection in males, as in *Th. entomophagus*. However, in females of *Th. ponticus*, setae *h*_3_ are only slightly longer than *ps_3_* (more than twice as long in *Th. entomophagus*), and the anal opening is approximately equal to the length of setae *h*_3_ (more than twice as long as the anal opening in *Th. entomophagus*). *Thyreophagus ponticus* is insufficiently described and is considered a species inquirenda [6]. 

*Thyreophagus italicus* Vacante, 1989 differs from *Th. entomophagus* by the shape of the base of the spermatheca, which is vase-shaped, narrow, with constriction (bell-shaped, wide, without constriction in *Th. entomophagus*), very short setae *w* III (long, filiform in *Th. entomophagus*), spiniform setae *u* and *v* III, the same length as *s* III (flattened, button-shaped, more than twice as short as *s* III in *Th. entomophagus*). 

*Thyreophagus entomophagus nominalis* is an insufficiently described taxon, considered a species inquirenda [6]. It differs from *Th. entomophagus* by having subequal setae *h*_1_ and *ps*_1_ in females [25] (*h*_1_ are distinctly longer than *ps*_1_ in *Th. entomophagus*).

Neotype. The need for typification of *Thyreophagus entomophagus* through neotype designation is based on the following: (i) several morphologically similar species are known (*Th. holda* sp. n., *Th. leclercqi*), but the differences between these nominal species are not well understood and may represent either interspecific or intraspecific variations; sequence data are lacking for these species; (ii) specimens from China identified as *Thyreophagus* “*entomophagus*” [27] (without morphological description) show uncorrected genetic distances (p-distance) of 18.09% at nucleotide level and 6.36% at the amino acid level (NC_066986 vs. OR640974), indicating that two genetically distinct species are involved. This suggests that previous re-descriptions of *Thyreophagus entomophagus* may not suffice for accurate identification of this species and a neotype is needed. Our proposed neotype will provide stability in the usage of the name *Thyreophagus entomophagus*, facilitating confident identification based on both morphology and DNA sequence data. To standardize the current usage of the name *Thyreophagus entomophagus*, we propose designating a neotype (female, accession #) from a commercial culture PBK20-0101-199.SM38 used for the industrial rearing of predatory mites as described here using light microscopy, SEM, and COX1 sequence data.

#### 3.2.3. *Thyreophagus holda* Klimov, Kolesnikov, Merckx, Duarte et Vangansbeke sp. n.

*Thyreophagus entomophagus* Fain et al., 2000: 154, Figure 1, Figure 2, Figure 3, Figure 4, Figure 5, Figure 6, Figure 7, Figure 8 and Figure 9 (heteromorphic deutonymph; misidentification).

Material. Adults and heteromorphic deutonymphs—culture 1, started from adult specimens collected in a sparrow nest in the suburban district of Berlin (Berlin Dahlem), W. Knülle [11]. 

Type material. Holotype (female, slide 29-90a, recognizable by unique shape on slide) and paratypes (six females, five males and four heteromorphic deutonymph) same data (slides 29-89, 29-89a, 29-90, 29-90a).

Depository. The holotype and 15 paratypes are deposited in the Royal Belgian Institute of Natural Sciences, Brussels, Belgium.

Etymology. Holda is a popular figure in German folklore.

**Figure 12:**
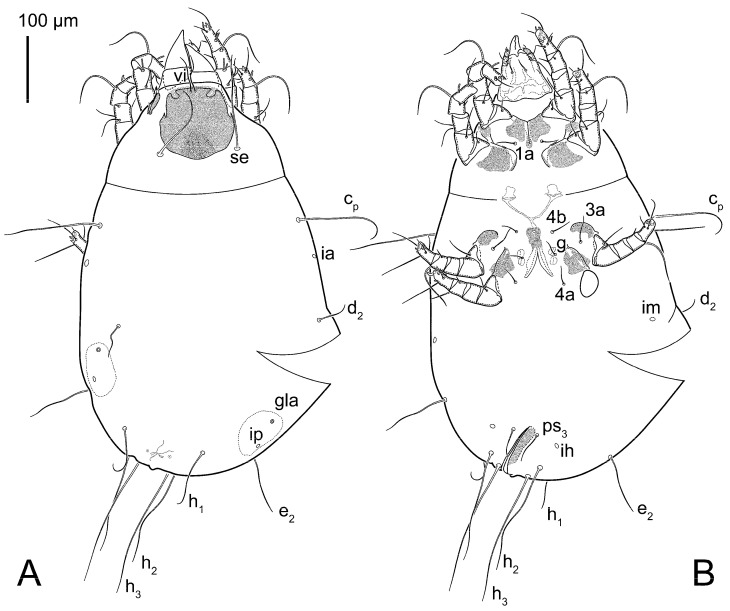
*Thyreophagus holda* sp. n., female, holotype: (**A**) dorsal view; (**B**) ventral view.

Female (n = 7) (Figure 12, Figure 14A–D,F,G, Figure 16A,B, Figure 17A,B,D–G,J and Figure 18B,E,I). Idiosoma slightly elongate, 580–800 × 300–400, 1.7–2.0 times longer than wide. Idiosomal cuticle smooth. Subcapitular setae (*h*) long, widened basally; palp tibial setae (*a*), lateral dorsal palp tibial setae (*sup*), dorsal palp tarsal seta (*cm*) filiform; supracoxal seta *elcp* present; terminal palp tarsal solenidion ω short; external part of terminal eupathidium *ul*” dome-shaped; terminal eupathidium *ul*’ small, rounded. Prodorsal shield 100–110 long, 110–112 wide, 1.0–1.1 times longer than wide, with setae *vi* (situated at anterior part of shield, alveoli noticeably separated—distance between them almost equal to their width), rounded anterolateral incisions, and elongate midlateral incisions (insertion points of setae *ve*). Prodorsal shield smoothly punctate (small cells), with lineate cells in posterior part and with several (3–5) curved longitudinal lines in posterior part. Grandjean’s organ (GO) with seven membranous finger-shaped processes. Supracoxal setae (*scx*) smooth, sword-shaped, widened and flattened, tapering at tip. Idiosomal setae (*vi*, *c*_p_, *d*_2_, *e*_2_, *h*_1_, *h*_3_, *ps*_3_) smooth, filiform and short, setae *se* and *h*_2_ twice as long as other idiosomal setae, smooth and filiform; opisthosomal gland openings between setal bases *e*_2_ and *d*_2_. Four pairs of fundamental cupules (*ia*, *im*, *ip* and *ih*) present. With a pair of additional cuticular pores between setae *h*_1_. Ventral idiosoma with four pairs of coxal setae (*1a*, *3a*, *4a* and *4b*) and one pair of genital setae (*g*). Shape of coxal sclerites as in Figure 12B. Genital region situated between coxal fields III and IV; genital valves form an inverted Y; epigynal and medial apodemes well-developed. Diameter of genital papillae approximately 0.4–0.5 the length of coxal and genital setae. Anal opening terminal. Copulatory tube present, situated anterodorsally to anus, with developed opening. Inseminatory canal of spermatheca long, slender tube-like, leading from copulatory opening to spermatheca, slightly widened at junction with base of spermatheca. Base of spermatheca wide, bell-shaped, with a distinct vestibule. Paired efferent ducts elongated, their length is approximately 1/2 the length of the base of spermatheca, with short stem.

Legs short, all segments free. Trochanters I–III each with long, filiform seta, *pR* I–II, *sR* III; trochanter IV without setae. Femoral setation 1-1-0-1; setae *vF* I–II and *wF* IV long, filiform. Genual setation 2-2-0-0; setae *mG* and *cG* I–II long, filiform; seta *nG* III absent. Tibial setation 2-2-1-1; setae *hT* I-II spiniform, shorter than *gT*; setae *gT* I–II and *kT* III–IV elongate, somewhat spiniform. Tarsal setation 8-8-8-8; pretarsi consist of hooked empodial claws attached to short paired condylophores. Tarsus I and II with setae *ra*, *la*, *f* and *d* filiform, *e*, *u*, *v* spiniform, *p* and *q* represented by very small remnants, *s* flattened, button-shaped or minute spiniform; setae *wa* absent. Tarsus III and IV with setae *f*, *d*, *r*, *w* filiform, *e*, *s*, *u* and *v* spiniform (*u* and *v* slightly less than *s*), *p* and *q* represented by very small remnants. Solenidion ω_1_ on tarsus I cylindrical, with clavate apex, in front of which there is a slight narrowing, curved; solenidion ω_1_ on tarsus II simple, cylindrical, with clavate apex, not bent. Solenidion ω_2_ on tarsus I shorter than ω_1_, cylindrical, with rounded apex, slightly widened distally, situated slightly distal to ω_1_. Solenidion ω_3_ on tarsus I cylindrical, with rounded tip, subequal to ω_1_, longer than ω_2_. Famulus (ε) of tarsus I wide, spiniform, with broadly rounded apex, widest at middle. Solenidia ϕ of tibiae I–III elongate, tapering, extending well beyond apices of respective tarsi with ambulacra; solenidion ϕ IV shorter, shorter than tarsus IV (with ambulacra). Solenidia σ’ and σ” on genu I elongate, tapering, σ” longer than σ’, σ” slightly not reaching bases of ϕ I. Solenidion σ on genu II more than 6–7 times longer than its width) with rounded tip. Solenidion σ of genu III absent.

**Figure 13:**
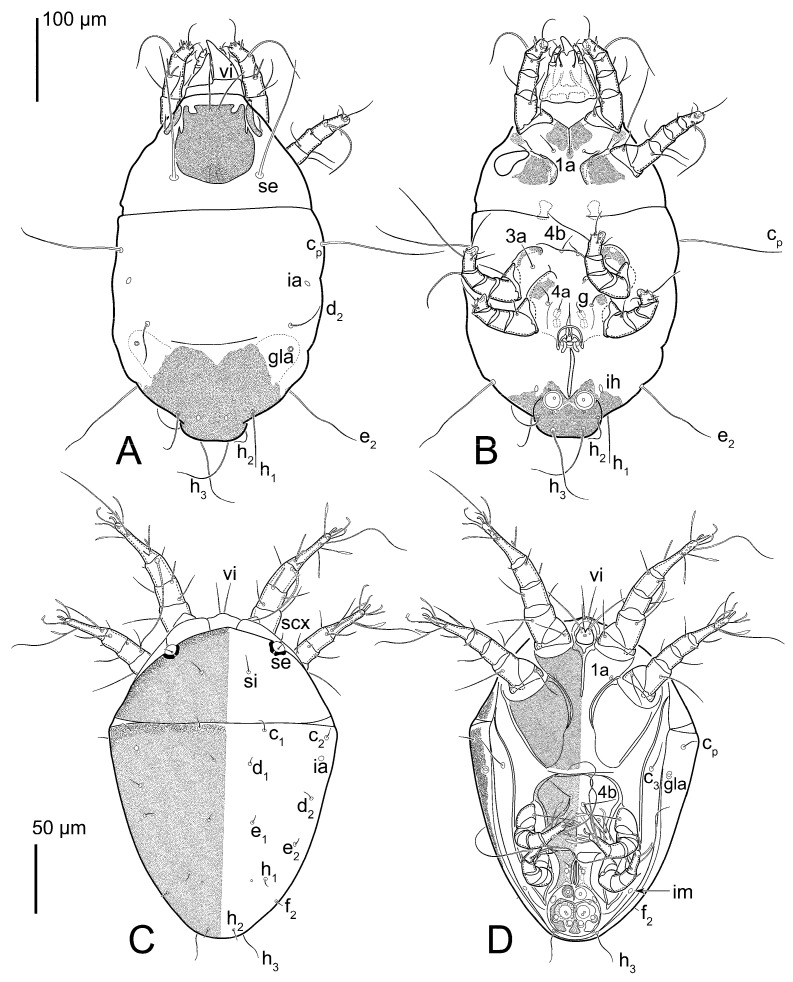
*Thyreophagus holda* sp. n., paratypes: (**A**) male, dorsal view; (**B**) male, ventral view; (**C**) heteromorphic deutonymph, dorsal view; (**D**) heteromorphic deutonymph, ventral view.

**Figure 14:**
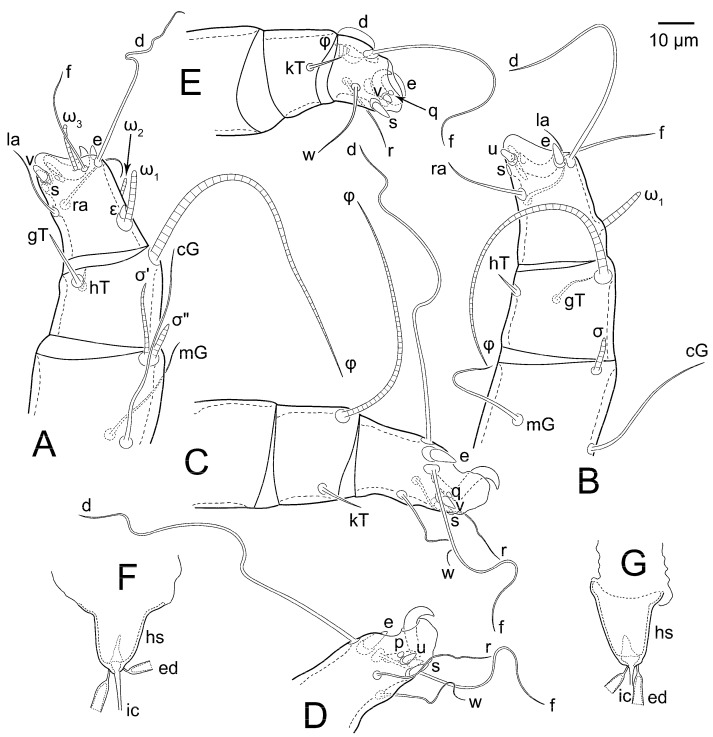
*Thyreophagus holda* sp. n., holotype (**A**–**D**,**F**) and paratypes (**E**,**G**): (**A**) female leg I, posterior view; (**B**) female leg II, anterior view; (**C**) female leg III, anterior view; (**D**) female tarsus III, posterior view; (**E**) male leg IV, anterior view; (**F**,**G**) spermathecae.

Male (n = 5) (Figure 13A,B, Figure 14E, Figure 16C,D and Figure 17C,H,I). Idiosoma slightly elongate, 430–470 × 250–300, 1.6–1.7 times longer than wide. Idiosomal cuticle smooth. Gnathosoma as in female. Prodorsal shield 86–95 long, 90–98 wide, 1.0–1.1 times longer than wide, with setae vi, incisions and ornamented as in female. Grandjean’s organ (GO) and supracoxal seta (*scx*) as in female. All idiosomal setae smooth and filiform, setae *se* linger and widener that other, setae *c*_p_, *e*_2_ and *h*_3_ longer than *vi*, *d*_2_, *h*_1_, *h*_2_. Two pairs of fundamental cupules (*ia* and *ih*) present, *im* and *ip* not observed. Between setae *h*_1_, there is a pair of additional cuticular pores. Opisthonotal shield smoothly punctate; ventral part extends to anal suckers. Ventral idiosoma with four pairs of coxal setae (*1a*, *3a*, *4a* and *4b*) and one pair of genital setae (*g*). Posterior region of idiosoma with a large rounded lobe extending the body backwards (45–50 × 78–85, 1.5–1.8 times longer than wide). Shape of coxal sclerites on Figure 13B. Genital region between coxisternal fields IV; arms of genital capsule rounded; aedeagus short, not protruding beyond anterior edge of genital capsule. Diameter of genital papillae approximately 0.4–0.5 the length of coxal and genital setae. Anal suckers rounded in outline. Setae *ps*_1–3_ very short.

Legs I-III as female, except solenidion ω_3_ on tarsus I very short, truncated. Trochanter and genu IV without setae, femur IV with *wF* IV long, filiform, tibia IV with *kT* IV elongate, somewhat spiniform. Tarsus IV with 8 setae, of them, *f*, *r*, *w* filiform, *d* and *e* represented by suckers, *s*, *e*, *u* and *v* spiniform (*u* and *v* slightly less than *s*), *p* and *q* represented by very small remnants. Solenidion ϕ on tibia IV short and wide.

**Figure 15:**
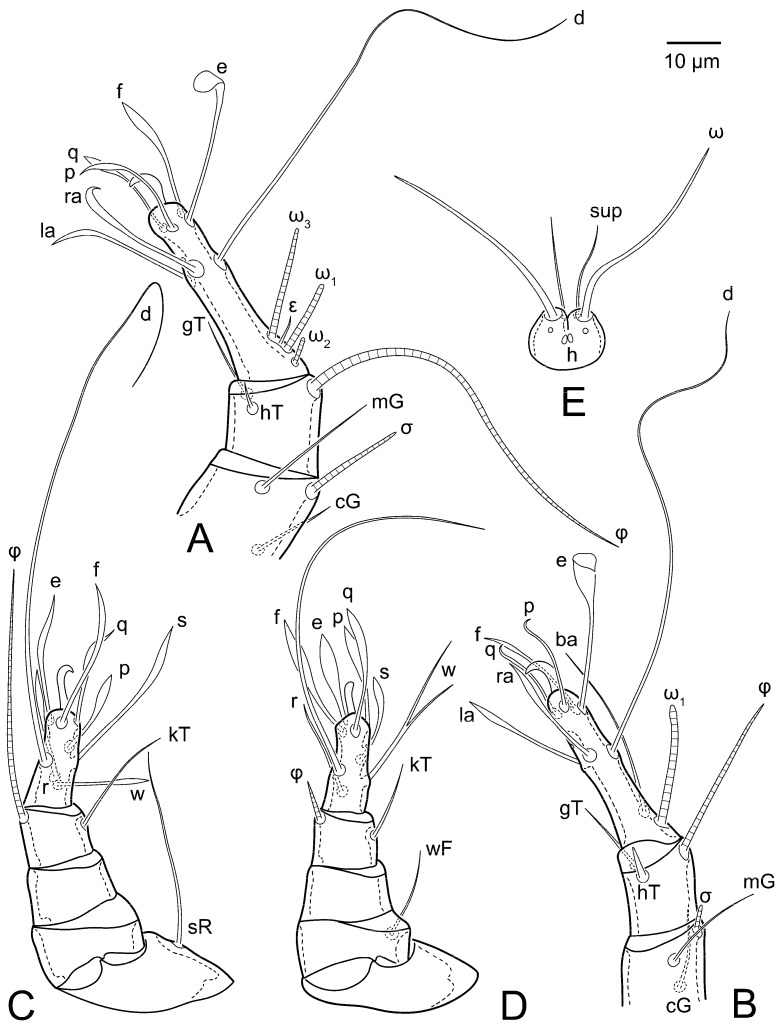
*Thyreophagus holda* sp. n., heteromorphic deutonymph, paratype: (**A**) leg I, antiaxial view; (**B**) leg II, anterior view; (**C**) leg III, anterior view; (**D**) leg IV, anterior view; (**E**) gnathosoma, ventral view.

**Figure 16:**
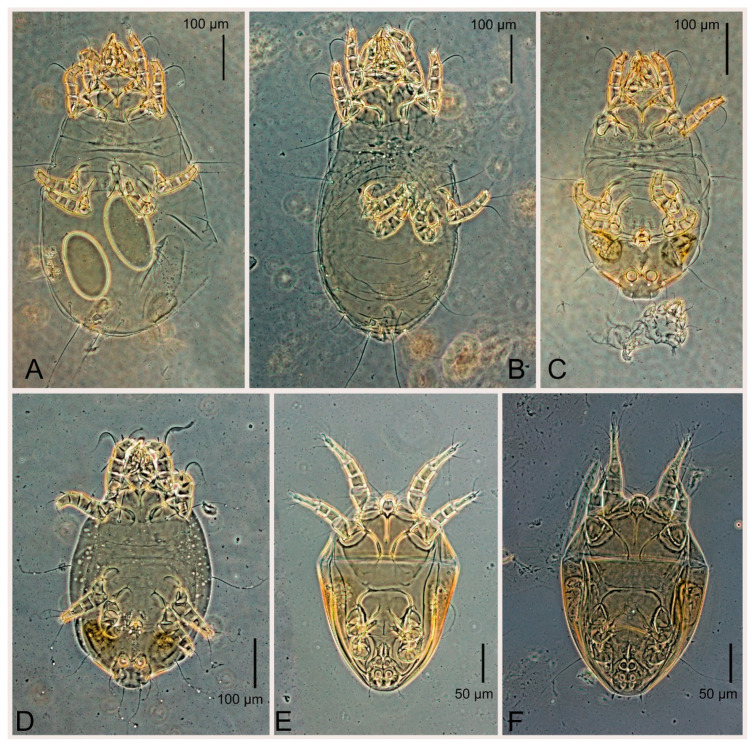
*Thyreophagus holda* sp. n., phase contrast images: (**A**) female holotype; (**B**) female paratype; (**C**,**D**) male paratypes; (**E**,**F**) heteromorphic deutonymph paratypes.

**Figure 17:**
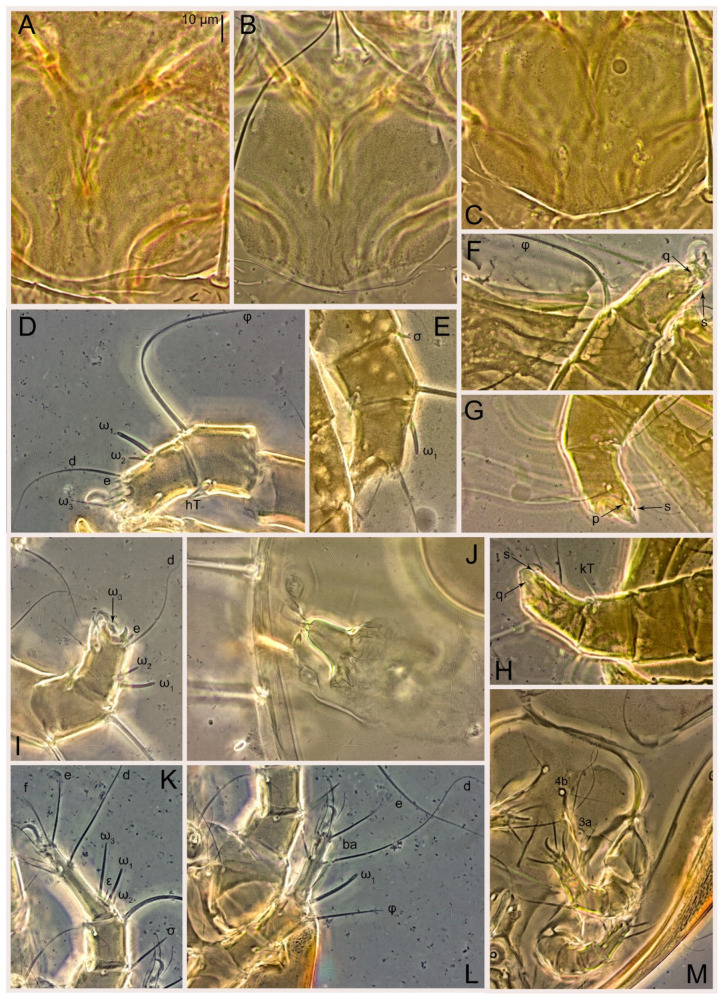
*Thyreophagus holda* sp. n., holotype (**A**,**F**,**J**) and paratypes (**B**–**E**,**I**,**H**–**M**), phase contrast images: (**A**,**B**) female prodorsal shields; (**C**) male prodorsal shield; (**D**) female leg I, anterior view; (**E**) female leg II, posterior view; (**F**) female leg III, posterior view; (**G**) female leg III, anterior view; (**H**) male leg III, posterior view; (**I**) male leg I, anterior view; (**J**) spermatheca; (**K**) heteromorphic deutonymph leg I, anterior view; (**L**) heteromorphic deutonymph leg II, anterior view; (**M**) heteromorphic deutonymph legs III–IV.

Heteromorphic deutonymph (n = 4) (Figure 13C,D, Figure 15, Figure 16E,F and Figure 17K–M). Body ovoid, 1.3–1.4 times longer than wide, widest in sejugal region; idiosomal length 240–250 width 170–190. Gnathosoma short, subcapitulum and palps fused, bearing palpal solenidia (ω) apically and filiform apicodorsal setae (*sup*); setae *h* absent (their positions marked by somewhat refractile spots), setae *cm* absent.

Dorsum. Idiosoma smoothly punctate; distinct linear pattern present on anterior and lateral sides of prodorsal sclerite and hysterosomal shield. Apex of propodosoma anterior to anterior border of prodorsal sclerite, with apical internal vertical setae (*vi*) (bases separated) and a pair of band-like sclerites coalescing anteriorly. A pair of lateral, widely separated ocelli (distance 68–70) present on prodorsum; lenses and pigmented spots present; maximum diameter of lenses 9–10. External vertical setae (*ve*) absent; external scapular setae *se* situated below lenses; internal scapular setae (*si*) distinctly posterior external scapulars (*se*). Supracoxal setae of legs I (*scx*) filiform, with extended base, situated below *si* and anterolaterad of ocelli. Sejugal furrow well developed. Prodorsal sclerite 70–75, hysterosomal shield 158–173, ratio hysterosomal shield/prodorsal sclerite length = 2.2–2.3. Hysterosoma with 11 pairs of simple, filiform setae on hysterosomal shield (*c*_1_, *c*_2_, *c*_p_, *d*_1_, *d*_2_, *e*_1_, *e*_2_, *f*_2_, *h*_1_, *h*_2_, *h*_3_), setae *h*_3_ distinctly longer than other setae. Opisthonotal gland openings (*gla*) situated ventrally on hysterosomal shield, slightly posterior setae *c*_3_, much closer to ventral seta *c*_3_ than to dorsolateral seta *c*_p_ (in one specimen approximately equidistant from setae *c*_3_ and *c*_p_). Of four fundamental pairs of cupules, three pairs were observed: *ia* posteriomediad setae *c*_2_, *im* ventral, laterad trochanters IV and *ih* ventral, laterad posterior sides of attachment organ.

Venter. Coxal fields sclerotized, smoothly punctate. Anterior apodemes of coxal fields I fused forming sternum; sternum not reaching posterior border of sternal shield by distance exceeding its length. Posterior border of sternal shield not sclerotized. Anterior apodemes of coxal fields II curved medially. Posterior apodemes of coxal fields II weakly developed, thin, curved medially. Sternal and ventral shield contiguous. Anterior apodemes of coxal fields III free, connected by thin transverse sclerotization. Posterior medial apodeme present in area of coxal fields IV, well-separated from anterior apodemes IV and genital opening. Posterior apodemes IV absent. Subhumeral setae (*c*_3_) filiform, situated on ventral surface between legs II and III, near region separating sternal and ventral shields. Coxal setae *1a* and *3a* reduced, represented by minute structures. Setae *4b*, *g* filiform; *4a* in form of small, rounded conoids. Genital region in posterior portion of coxisternal fields IV; genital opening elongate, with two pairs of genital papillae within genital atrium; genital papillae two-segmented, with rounded apices. Coxal setae (*4b*) situated at free ends of anterior coxal apodemes IV; genital setae (*g*) laterad genital opening. Attachment organ posterior to coxal fields IV. Anterior suckers (*ad*_3_) round, median suckers (*ad*_1+2_) distinctly larger, with paired vestigial alveoli (not situated on common sclerite); pair of small refractile spots anterolaterad median suckers (*ps*_3_); lateral conoidal setae of attachment organ (*ps*_2_) situated distinctly posterior to line joining centers of median suckers, slightly anteriad conoidal setae (*ps*_1_); anterior and posterior lateral and posterior median cuticular conoids well developed; anus situated between anterior suckers (*ad*_3_).

Legs. Legs elongate, all segments free. Trochanters I–III each with long, filiform seta, *pR* I–II, *sR* III. Femoral setation 1-1-0-1; setae *vF* I–II and *wF* IV long, filiform. Genual setation 2-2-0-0; setae *mG* and *cG* I–II filiform, seta *nG* III absent. Tibial setation 2-2-1-1; setae *hT* I somewhat spiniform; setae *gT* I-II filiform; setae *hT* II spiniform, setae *gT* longer than *hT*; setae *kT* III-IV filiform, without a prong. Tarsal setation 8-9-8-8. All pretarsi consisting of hooked empodial claws attached to short paired condylophores. Tarsus I with setae *ra*, *la*, *p*, *q*, *e*, *f* foliate; seta *d* filiform, its base at level of *ra* and *la*; seta *s* represented by alveolus; setae *wa*, *aa* and *ba* I absent; tarsus II similar to tarsus I except seta *ba* present, filiform, situated close to ω_1_. Tarsus III with setae *w*, *r*, *s*, *p*, *q*, *e*, *f* and *d* smooth; all setae, except for *d* III, more or less foliate; seta *d* longer than leg. Tarsus IV similar to tarsus III, except seta *r* filiform and *w* filiform with a distinct prong. Solenidia ω_1_ on tarsi I–II cylindrical, with slightly clavate apices; ω_3_ on tarsus I longer than ω_1_, with rounded apex, situated slightly distal to ω_1_; ω_1_ and ω_3_ separated by bulbous famulus (ε); solenidion ω_2_ of tarsus I slightly expanding apically, situated somewhat more basal and posterior to ω_1_ + ε + ω_3_ group; solenidia φ of tibiae I–III elongate, tapering; φ I and III longer than tarsus I and III, respectively; solenidion φ II shorter than tarsus II; solenidion φ IV short; solenidion σ of genu I elongate, slightly tapering, nearly reaching tip of tibia I; solenidion σ of genu II much shorter, cylindrical, not reaching midlength of tibia II; solenidion σ of genu III absent.

**Figure 18:**
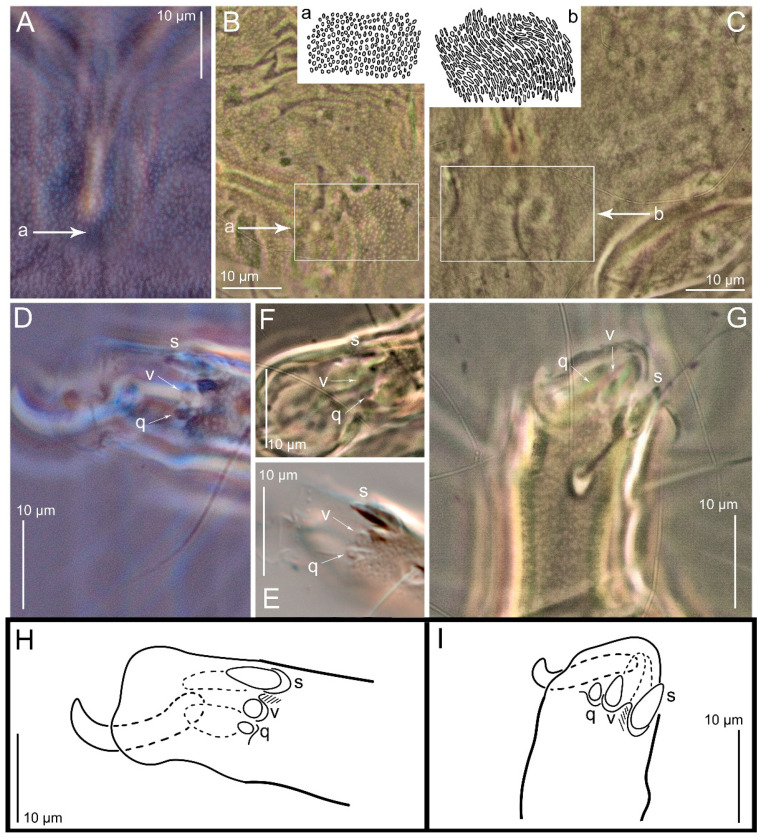
*Thyreophagus entomophagus* (Laboulbène and Robin, 1862) (**A**,**B**,**D**–**F**,**H**), PBK20-0101-199.SM38, not neotype (**A**,**D**,**E**,**H**), Birmingham specimens (**B**,**F**) and *Th. holda* sp. n. (**C**,**G**,**I**), phase contrast images (**A**–**D**,**F**,**G**), DIC images (**E**) and lineal drawings (**H**,**I**) distinctive morphological characteristics: (**A**–**C**) punctated posterior part of female prodorsal shields; (**D**–**F**) female tarsi III with flattened, button-shaped setae *v*, anterior view; (**G**) female tarsi III with spiniform setae *v*, anterior view. Abbreviations: a—rounded and elongated cells; b—lineate cells; *s*, *v*, *q*—tarsal ventral setae.

Diagnosis. Adults of *Th. holda* are very similar to those of *Th. entomophagus*, but differ by the following: apical tarsal setae *u* and *v* III-IV normally developed, spiniform (vs. flattened and button-shaped in *Th. entomophagus*) (Figure 18G,I vs. Figure 18D–F,H); prodorsal shield with a lineate cells in its posterior part (vs. rounded or slightly elongated in *Th. entomophagus*) (Figure 18C vs. Figure 18A,B); larger body measurements (females 580–800 and males 430–470 vs. females 400–430 and males 300–360 in *Th. entomophagus*). The new species forms heteromorphic deutonymphs (vs. lacking in *Th. entomophagus*).

Heteromorphic deutonymphs of *Th. holda* sp. n. are similar to *Th. leclercqi* (Fain, 1982) [28] and *Th. australis* (Clark, 2009) [29] in terms of their ovoid bodies, which are 1.3–1.4 times longer than they are wide, compared to the elongate bodies (more than 1.7 times longer than they are wide) in other species. *Th. holda* differs from *Th. leclercqi* by their short setae *hT* I, which are more than 2 times shorter than *gT* I (less than 2 times shorter in *Th. leclercqi*). *Th. holda* differs from *Th. australis* by their setae *kT* III, which lack a prong (with a distinct prong in *Th. australis*), and the absence of setae *wa* I-II (present in *Th. australis*).

Based on Figure 1 in reference [11], Barbosa et al. [30] and Klimov et al. [5] suggested that one of the diagnostic character states of *Th. holda* sp. n. heteromorphic deutonymphs is the position of the opisthonotal gland openings, which are approximately equidistant from setae *c*_3_ and *c*_p_. However, our study showed that only one specimen of *Th. holda* sp. n. shows this character state, while the remaining specimens from the same culture have the usual arrangement of these openings (*gla* is much closer to ventral seta *c*_3_ than to dorsolateral seta *c*_p_). Based on these findings, we suggest that this character is variable and should not be considered diagnostic for *Th. holda* sp. n.

## 4. Discussion

The mite *Thyreophagus entomophagus* is an economically important species widely used as factitious prey in the industrial rearing of phytoseiid mites for biocontrol applications [1,5,6,7,31]. This is a cosmopolitan species [32] that has been reported in the literature from various habitats: entomological collections [9,10,33]; flour [34,35,36,37,38,39,40,41]; dry Spanish fly *Lytta vesicatoria* harvested for medicinal use [35]; vanilla pods and saffron for medicinal use [42]; medicinal plants, including ergot and cardamom; stimulants; spices; food products and fodders (rye and wheat bran) [33]; poultry meal [43]; on ergot of rye [42]; in bird nests [33,43]; bracket fungi *Fomitopsis betulina* [44]; and various materials in beehives of *Apis mellifera* [45,46] in association with scale insects [36,47,48], coccids [48], and trogid beetles [49].

However, the reliability of this name may be compromised due to several factors: the absence of original type specimens, difficulties in interpreting the classical species diagnosis by A. Fain [10], which is not entirely accurate with respect to actual specimens (e.g., the shape of ventroterminal setae *u* and *v* III–IV), the presence of closely related species such as *Th. holda* and *Th. leclercqi*, and the misidentified and divergent GenBank sequence that lacks accompanying morphological information. As a result, previous reports in the literature require verification. Here, to standardize the usage of the name *Thyreophagus entomophagus*, we propose designating a neotype for which both detailed morphological and sequence data are available. 

Our research confirms that *Th. entomophagus* lacks the heteromorphic deutonymph stage (Figure 1), a trait consistently observed in laboratory populations cultured independently across multiple labs and biocontrol facilities (our observations). Earlier reports from Germany indicated the presence of heteromorphic deutonymphs [11], but our study did not corroborate this, suggesting that the German population represents a different species, *Th. holda sp. n*. The absence of heteromorphic deutonymphs is advantageous for mass production, as it simplifies the life cycle by eliminating an energetically costly developmental stage. These deutonymphs are also heavily sclerotized, making them less palatable to predators. However, *Th. entomophagus* remains a sexual species, which is a less desirable trait for mass production since asexual species reproduce more quickly, generating only females. The only known species that is both asexual and lacks the heteromorphic deutonymph stage is *Th. plocepasseri* [22]. 

Our preliminary phylogenetic analysis suggests that the two traits important for biocontrol—asexual reproduction and direct development (i.e., absence of the heteromorphic deutonymph stage)—are derived, having emerged after the most recent common ancestor of *Thyreophagus* (Figure 2). While speculative, asexual reproduction may provide an advantage in subcortical habitats like fallen branches, where predator activity is low and the “Red Queen” dynamics [50,51] are absent. The lack of heteromorphic deutonymphs might also confer an advantage in vertebrate nests, where animals can transport branches, facilitating long-distance dispersal. Short-distance dispersal, such as within a nest, can be accomplished by the mites themselves, particularly the more mobile immature stages. In contrast, inseminated females tend to burrow into tunnels where they produce large numbers of eggs as slow-moving, ovigerous individuals. 

Our study not only standardizes the nomenclature of *Thyreophagus entomophagus*, a species widely utilized in the rearing of predatory mites, but also proposes identifying species that combine advantageous traits, such as asexual reproduction and a direct life cycle, for further bioprospecting efforts.

## 5. Conclusions

The mite *Thyreophagus entomophagus* is a cosmopolitan mite species with significant economic importance in biocontrol applications. Its diverse habitat range and frequent association with various food products and environments suggest its great adaptability. However, the taxonomic status of this species name is questionable due to inconsistencies in historical species identifications, the absence of original type specimens, and misidentified GenBank sequences. To address these issues and to standardize the nomenclature, we propose the designation of a neotype with both morphological and genetic data available. 

Our research shows that *Th. entomophagus* lacks the heteromorphic deutonymph stage, an advantageous trait for mass production, streamlining its life cycle. Nonetheless, this is a sexual species, a feature less desirable for mass-rearing programs. Phylogenetic evidence suggests that asexuality and the absence of heteromorphic deutonymphs are derived traits, likely advantageous in specific ecological niches like subcortical habitats or vertebrate nests. These findings offer important insights into the evolutionary traits that improve the effectiveness of *Th. entomophagus* and related species in biocontrol settings. Our study specifically suggests the need for additional bioprospecting efforts to find new candidate species for biocontrol applications that exhibit both asexual reproduction and the absence of heteromorphic deutonymphs.

## Data Availability

Data are available in a publicly accessible repository.
